# *In vitro* assays for clinical isolates of sequence type 131 *Escherichia coli* do not recapitulate *in vivo* infectivity using a murine model of urinary tract infection

**DOI:** 10.1128/spectrum.01511-24

**Published:** 2025-02-25

**Authors:** Courtney P. Rudick, Rachel S. Cox, Travis J. Bourret, Nancy D. Hanson

**Affiliations:** 1 Department of Medical Microbiology and Immunology, Creighton University School of Medicine, Omaha, Nebraska, USA; 2 Creighton Center for Antimicrobial Resistance and Epidemiology, Creighton University School of Medicine, Omaha, Nebraska, USA; Michigan State University, East Lansing, Michigan, USA

**Keywords:** *Escherichia coli*, sequence type 131, clinical isolates, *in vitro *virulence assays, mouse model, urinary tract infection, cystitis, pyelonephritis

## Abstract

Sequence type 131 isolates are a major cause of cystitis and pyelonephritis. Many studies rely solely on *in vitro* assays to screen for bacterial virulence factors associated with the pathogenicity of clinical isolates of *Escherichia coli*. Few studies have compared *in vitro* findings with *in vivo* infectivity of clinical isolates. The purpose of this study was to evaluate the correlation between *in vitro* assays with the ability to cause cystitis and pyelonephritis in a murine model of urinary tract infection. *In vitro* assays were conducted according to the published protocols and included motility assays, biofilm formation, epithelial cell adhesion and invasion, and curli production. Twenty-one uropathogenic *E. coli* (UPEC) isolates of *E. coli* ST131 and non-ST131 were used for both *in vivo* and *in vitro* studies. Six mice per isolate were inoculated via urethral catheterization. Colony forming units (CFUs) were determined from bladder and kidneys. *In vitro* and *in vivo* correlations were evaluated by multiple linear regression analysis. Pairwise linear regressions showed trendlines with weak positive correlations for motility, adhesion, and invasion and weak negative correlations for hemagglutination, biofilm, and curli production. The ability of *E. coli* ST131 and non-ST131 clinical isolates to cause cystitis and pyelonephritis varied among strains. The *R*^2^ Pearson correlation value was less than ±0.5 for any pair, indicating little to no statistical association between *in vitro* and *in vivo* findings. These data show that *in vitro* data are not predictive of the ability of ST131 *E. coli* to infect and/or cause disease in a mouse model.

**IMPORTANCE:**

Urinary tract infections (UTIs) affect 150 million people annually, and *E. coli* ST131, a pandemic clone, has become responsible for a significant portion of those UTIs. How ST131 *E. coli* has become such a successful strain remains to be elucidated. When evaluating bacterial pathogenicity, it is customary to use *in vitro* assays to predict isolate virulence and fitness due to lower cost and ease of experimentation compared with *in vivo* models. It is common to use model organisms like pathogenic *E. coli* CFT073 or a non-pathogenic K12 lab strain as representatives for the entire species. However, our research has shown that model organisms differ from ST131 *E. coli,* and *in vitro* assays are poor predictors of ST131 isolate infectivity in a murine model of UTI. As such, research into the mechanisms of fitness/pathogenesis for ST131 infectivity needs to focus on these organisms rather than other types of UPEC.

## INTRODUCTION

Urinary tract infections (UTIs) are the second most common bacterial disease occurring in 150 million people globally every year, with women between the ages of 18 and 24 years, and women over the age of 60 years at especially high risk of recurrent UTI (1, 2). Each year, one in five women in the most at-risk age group will have a symptomatic UTI; of these infections, *Escherichia coli* is the most common causative agent, being detected in over 75% of cases (1, 3, 4). *E. coli* that causes UTIs are categorized as uropathogenic *E. coli* (UPEC), and pathotypes associated with cystitis, pyelonephritis, and bacteremia are categorized as extraintestinal pathogenic *E. coli* (ExPEC) (3).

In order to colonize the urinary tract, *E. coli* must be able to evade host defenses, move from the intestinal tract to the periurethral opening, and adhere to the epithelial cells in the urethra, bladder, and kidney (5, 6). In addition, 4–24 h after bladder colonization, the bacteria express adhesins including curli, type I, and P-fimbriae (7). Type I fimbriae attach to the mannose moieties of uroplakin receptors expressed on the urethral epithelial cells, which allows the *E. coli* to travel up the urethra to the bladder (7). Cystitis occurs when ExPEC infection is localized to the bladder and is characterized by the continual expression of fimbriae by *E. coli* (7). If fimbriae are no longer expressed, the bacteria are able to ascend up the ureters and into the kidneys inducing pyelonephritis (7). Once in the kidneys, P-fimbriae attach to digalactoside receptors expressed on the kidney epithelial cells establishing infection (7). Virulence factors have been associated with certain functions such as the ability to attach to uroepithelium cells (such as type 1 fimbriae), biofilm formation (curli fibers) as well as host-immune evasion, and intracellular invasion of epithelial cells (8). Most studies evaluating pathogenicity in *E. coli* have evaluated the contribution of these mechanisms using *in vitro* assays including motility, biofilm production, mannose-binding assays, and cellular adhesion.

A major pathogenic clone in cystitis and pyelonephritis is *E. coli*, sequence type (ST) 131. This ExPEC pandemic clone was originally discovered in three continents (Asia, Europe, and North America) in 2008 (4, 6, 9). ST131 strains are multidrug-resistant and harbor chromosomal and plasmid-encoded resistance mechanisms to antibiotics used to treat UTIs including fluoroquinolones and β-lactams. The majority of data in the literature evaluate ST131 *E. coli* using whole genome sequencing, identifying genes involved in resistance and/or virulence. However, as shown for other UPEC isolates, no single set of virulence factors can be attributed to the success of ST131 isolates (10–14). Whole genome sequencing data, although useful, are insufficient to predict the pathogenicity of *E. coli* ST131. Several murine UTI models have been established and used to evaluate different strains of UPEC isolates (15–17). However, animal studies used to evaluate the pathogenicity of ST131 have mostly used sites other than the urinary tract (i.e., peritoneal injections) for infection (18–20). In addition, studies have used *in vitro* assays to help determine the potential pathogenicity of a particular strain (21, 22). We hypothesized that *in vitro* evaluation of the mechanisms associated with pathogenicity may not predict the ability of isolates to infect and cause disease using a mouse UTI model. To test our hypothesis, we characterized 21 clinical UPEC isolates of *E. coli* including 15 ST131, and six non-ST131 in both *in vitro* and *in vivo* systems to determine the correlation between these two types of analyses. Unlike studies that evaluate pathogenesis/fitness using either *in vitro* or *in vivo* methods, we evaluated the same isolates using both experimental approaches. Surprisingly, little or no correlation was observed in this study between the isolates that were successful in causing a bladder and/or kidney infection, and the results from *in vitro* assays commonly used to measure mechanisms associated with infectivity and pathogenicity.

## RESULTS

### Strains used in this study

The characteristics of the strains used in this study are provided in Table 1. Characteristics include sequence type, phylotype, geographical location of collection, the β-lactamase produced by the strains, and the susceptibilities to a range of antibiotics. In order to compare the isolates used in this study, we first determined if there were any fitness differences among the strains. Growth curves were performed, and no difference in the time required to reach mid-log growth among the isolates was observed (Fig. S1A; Table S1). Strain CFT073 (ST73) was the control UPEC for *in vitro* assays (22). Knockout strains of BW25133 (BW), BWΔ*ompC* and BWΔ*ompF*, were acquired from the Keio Collection, and the double knockout clone was generously provided by Dr Heike Brötz-Oesterhelt (23). Porins play an important role in antibiotic susceptibility. These knockout strains were used to assess the role of OmpC and OmpF in infectivity and/or their impact on *in vitro* assays.

**TABLE 1:** Properties of *E. coli* isolates used in this study^*a*^

					Zone diameter (mm)						
Strain	Sequence type	Phylotype	Geographical location	*bla*_CTX-M_ allele	Cefotaxime	Ampicillin	Gentamicin	Ciprofloxacin	Amikacin	Polymyxin B	Ertapenem
BW25113	10	A	Stanford, CA	None	28	16	22	28	19	14	33
BWΔo*mpC*	10	A	Stanford, CA	None	43	24	23	33	21	14	33
BWΔ*ompF*	10	A	Stanford, CA	None	33	14	21	31	24	14	33
BWΔo*mpC*Δ*ompF*	10	A	Stanford, CA	None	37	17	22	35	27	15	27
C14	648	D2	Omaha, NE	CTX-M-14	9	6	21	6	18	13	28
C15	405	B1	Omaha, NE	CTX-M-15	27	6	20	6	18	13	32
C15MP	405	B1	Omaha, NE	None	28	18	19	6	17	13	32
CFT073	73	B2	Baltimore, MD	None	31	19	22	30	17	13	32
CMB106	131	B2	Minneapolis, MN	CTX-M-15	6	6	8	6	26	13	27
CUMC247	131	B2	Omaha, NE	CTX-M-15	9	6	17	6	15	12	28
FO44	131	B2	United Kingdom	CTX-M-14	12	6	20	6	16	14	32
FHM6	131	B2	India	CTX-M-15	6	6	18	6	15	14	29
JJ2244	131	B2	Detroit, MI	CTX-M-15	6	6	23	6	17	13	27
La14	131	D2	Omaha, NE	CTX-M-14	13	6	6	10	18	13	29
Lo14	405	D2	Omaha, NE	CTX-M-14	8	6	6	6	18	15	28
MHHBS4	131	B2	Spain	CTX-M-15	6	6	20	6	18	13	27
MHVLab2	131	B2	France	CTX-M-15	6	6	6	6	15	14	27
NL217	131	B2	United Kingdom	CTX-M-14	12	6	18	6	18	13	29
RS007	131	B2	United Kingdom	CTX-M-15	13	6	17	6	15	13	30
RS059	131	B2	United Kingdom	CTX-M-15	6	6	22	6	17	13	28
RS135	131	B2	United Kingdom	CTX-M-15	6	6	8	8	17	13	34
W15	131	B2	Omaha, NE	CTX-M-15	6	6	20	6	17	13	29
XQ13	68	D	Seattle, WA	CTX-M-14	6	6	25	29	17	13	26
XQ35	131	B2	Seattle, WA	CTX-M-15	16	6	21	6	15	13	32
XQ12	131	B2	Seattle, WA	CTX-M-15	6	6	6	6	16	13	28

^*a*^ Table recreated with modifications from (24). BW strains are laboratory strains all other *E. coli* are clinical isolates. Cefotaxime Susceptible (S) (≥26), Intermediate (I) (23–25), Resistant (R) (≤22); Ampicillin S (≥17), I (14–16), R (≤13); Gentamicin S (≥15) I (13–14) R (≤12); Ciprofloxacin S (≥21), I (16–20), R (≤15); Amikacin S (≥17), I (15–16), R (≤14); Polymyxin B S (≥12), R (≤11); Ertapenem S (≥22), I (19–21), R (≤18). BWΔ*ompC*Δ*ompF* was a gift from the laboratory of Professor Brötz-Oesterhelt.

### Variable pathogenicity observed among ST131 *E. coli* in a UTI mouse model

A UTI mouse model was used to establish the infectivity and pathogenicity of different strains of *E. coli* ST131 compared with non-ST131 *E. coli*. Six mice were inoculated with each *E. coli* strain using an ascending urinary tract infection model. Urine was collected from the mice up to day 28, and mice were sacrificed; bladder and kidneys were removed and evaluated. Colony forming units (CFUs) were determined for the urine, and bladder and kidney tissues. All mice survived infection except for one. Mouse #2 in the W15 group died from peritoneal sepsis 36 h after inoculation.

**Fig 1:**
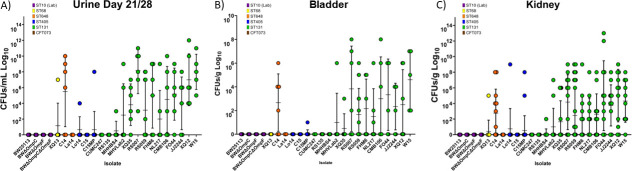
Urine colonization. CFUs from each mouse in the study on the final day of collection (A) urine, (B) bladder, and (C) kidney. Each data point represents the CFUs detected in the sample from a single mouse, and error bars represent the mean and standard deviations. Data point color represents the isolate sequence type: purple, ST10; yellow ST68; orange ST648; blue ST405; green ST131; brown CFT073.

To determine the bacterial load and ability of the isolates to cause a sustained UTI, urine was collected at several time points throughout the study before and after inoculation. At day 28, isolates XQ13, Lo14, and C15MP were detected in a single mouse per group, whereas C14 and most ST131 isolates (excluding CUMC247, MHHBS4, and RS135) were detected at high levels (up to 10^12^) in multiple mice at the end of the study (Fig. 1). No lab strains were detected in urine at the end of the study. Of note, XQ12 and W15 were detected in all mice in their respective groups throughout the course of the study (Fig. 1; Fig. S2A and Fig. S2B through E).

The CFUs for each isolate throughout the duration of the study are shown in Fig. S2A. In general, ST131 isolates caused more sustained infections with higher CFUs. Specific representative isolates have been highlighted in panels Fig. S2B through E. For example, Fig. S2B shows the infection with BWΔ*ompF* (ST10 Lab Strain) peaked at day 3 and was rapidly cleared. W15 (ST131) started with high CFUs and increased over the course of the study (Fig. S2C). C15 (ST405) had detectable CFUs that dropped off, returned on days 10 and 14, and then were cleared completely (Fig. S2D). C14 (ST648) increased steadily until day 10, and then leveled off until the end of the study (Fig. S2E).

At 28 days, mice were euthanized, and bladders and kidneys were collected, weighed, homogenized, serially diluted, and plated to determine the presence of infection and bacterial load per gram of the organ. The colored data points in Fig. 1 represent the *E. coli* bacterial load of each mouse bladder at day 28. None of the ST10 lab strains caused bladder infection at the end of the study. Only two non-ST131s were present in bladder tissue, C14 (ST648) and C15MP (ST405). C15MP had one positive mouse bladder with detectable, but low levels of inoculum. Most ST131 isolates were able to colonize bladder tissues at 10^3-8^ CFUs; however, ST131 strains CUMC247, RS135, and MHHBS4 were not detected in the bladder on the last day of the study. The most efficient ST131 for bladder infection was strain W15, which infected 100% of the mouse bladders with CFUs of 1 × 10^8^.

Individual mouse kidneys infected by the various strains are shown in Fig. 1. The colored data points represent the *E. coli* bacterial load of each mouse kidney at day 28. In addition to infections by C14 and C15MP, CFUs were present in the kidney for non-ST131 isolates XQ13 (ST68), Lo14 (ST405), and C15 (ST405). CFUs were also detected in the kidney for ST131 isolate MHHBS4. Interestingly, in strains where the number of colonized kidneys was low, the detectable CFUs are comparable between all infected tissues. Two mice in the RS135 group showed visible changes to kidney tissues with enlarged, clear fluid-filled, kidneys, but no bacteria were detected. As observed in the bladder, strain W15 infected the greatest number of kidneys (9/12) with CFUs of 1 × 10^9^. A side-by-side comparison of CFUs from all urine, bladder, and kidney samples can be found in Fig. 1.

### *In vitro* assessment of common mechanisms of fitness and virulence

*In vitro* assays used to evaluate pathogenicity of the 25 isolates included motility, biofilm production, mannose-binding assays, and cellular adhesion and invasion. For all the evaluations except motility, both static and shaking cultures were used. Published data have shown that subpopulations of both static and metabolically active cells are found in the bladder during an infection (25–28). Therefore, although these assays are typically done using only static cultures, we reasoned that in a urinary tract infection, it would be unlikely that the *E. coli* would exhibit only a static phenotype.

### Motility assays

**Fig 2:**
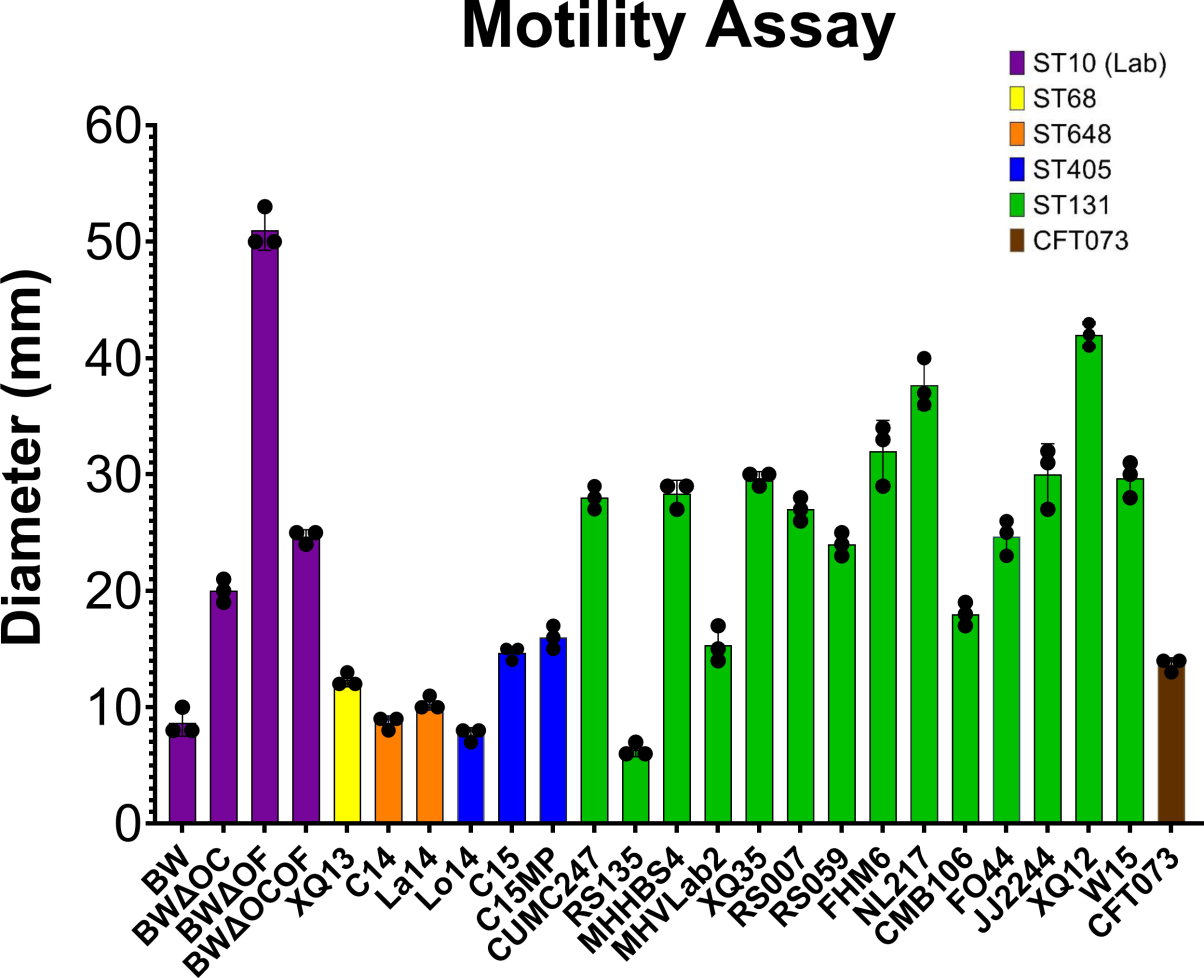
Motility of *E. coli* isolates. Zone of growth (measured across the diameter in mm) shows the ability of the isolate to move through semi-solid Agar (0.3% bactoagar in LB). Bars represent the mean diameter of three biological replicates indicated by data points and are colored according to the isolate sequence type as defined in the figure legend. Error bars are standard deviations. Images of individual plates can be found in Fig. S3.

Motility assays were conducted to evaluate flagella-mediated swimming motility (stab-inoculation into a soft agar plate). Motility as determined by zone diameter of migration is depicted in Fig. 2; Fig. S3. All of the strains tested were capable of movement but to varying degrees. Overall, the ST131 strains showed greater motility than non-ST131 strains (26 mm vs 17 mm, respectively, *P* = 0.00115). The non-ST-131 strains (ST68, ST648, and ST405) showed the least amount of motility among the strains tested and were equivalent to the laboratory strain, BW. Larger zone sizes were observed for most of the ST131 isolates, indicating the ability to move. Only one ST131 strain (RS135) showed a decrease in motility compared with the other ST131 isolates and was more closely related to the movement of non-131 strains. This isolate was also incapable of infecting the bladder and the kidney using the mouse model. The lab strains deficient in porin production of either OmpC (BWΔ*ompC*) or OmpF (BWΔ*ompF*) or the double knockout strain (BWΔ*ompC* Δ*ompF*) showed similar motility to the ST131 strains. Interestingly, the OmpF knockout, BWΔ*ompF*, had the largest zone size at 51 mm. All three *E. coli* knockout mutants showed increased motility compared with the parent strain BW (<10 mm). Of interest, the motility of the control strain CFT073 was similar to the non-131 strains and the laboratory strain BW but was not as motile as the ST131 strains. Although W15 was the most infective in the murine model, it only demonstrated average motility with XQ12, FHM6, NL217, and JJ2244 all being more motile while less infective.

### Biofilm formation

**Fig 3:**
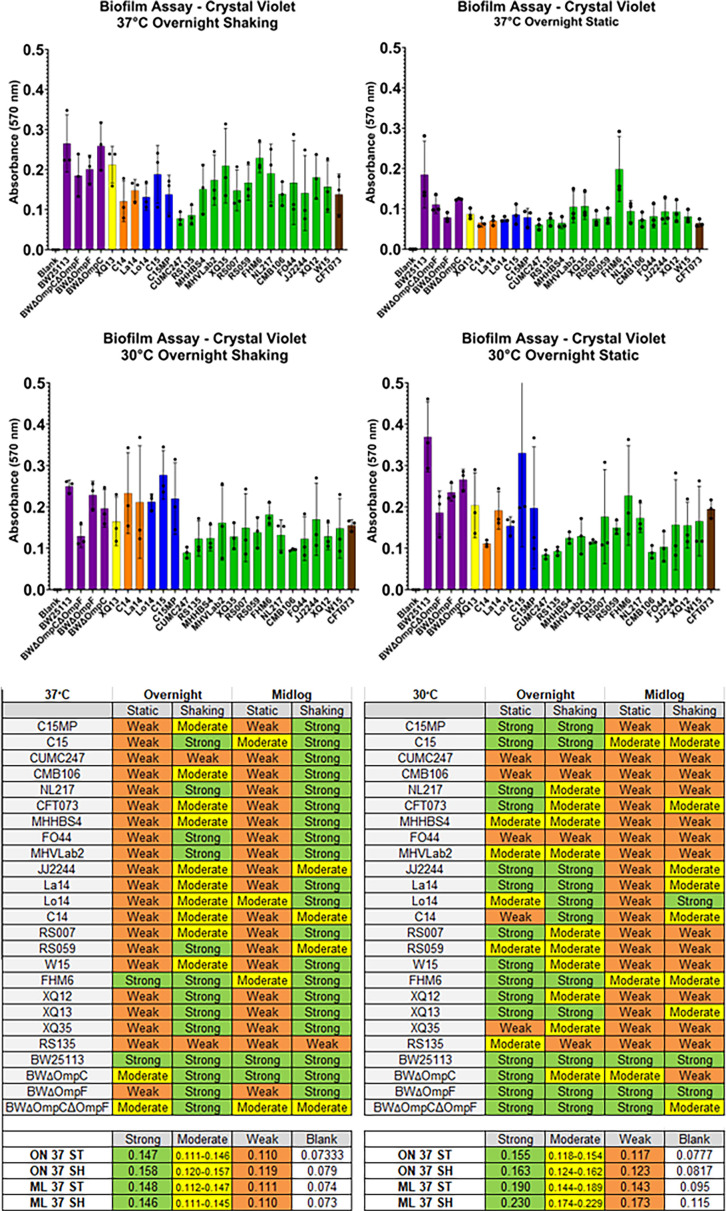
Biofilm production. Biofilm production was measured by crystal violet staining and O.D. Experiments were conducted under static and shaking conditions at both 37ᵒC and 30ᵒC. Isolate sequence type is noted by bar color; bars represent the mean absorbency of three biological replicates indicated by data points, and error bars represent standard deviations. The degree of biofilm production was classified according to the following criteria: the degree of biofilm production was classified according to the following criteria: Strong (OD > 2 × ODc), moderate (1.5 × ODc < OD≤2 × ODc), weak (ODc < OD ≤ 1.5×ODc), or absent (OD ≤ ODc) were coded as previously described (30). Values for each classification are listed at the bottom of the chart.

Biofilm formation is often associated with antibiotic resistance and can be considered a mechanism that contributes to pathogenicity. Although biofilm production is typically performed using static culture conditions at 30°C, we evaluated biofilm production using both static or shaking conditions at both 30°C and 37°C, as 37°C is more physiologically relevant (29). In general, less biofilm was produced in all isolates under static conditions at 37° compared with 30°C (Fig. 3). Interestingly, shaking conditions at 37°C showed more biofilm formation than static cultures; however, this was not the case at 30°C. (Fig. 3). When the cells were grown to midlog, the static conditions yielded much less biofilm formation for all isolates tested (Fig. S4). When midlog cells were evaluated at 30°C, there was little difference between shaking and static conditions (Fig. S4 and S5). In most conditions, biofilm production was highest for the ST10 lab strains with the exception of BWΔ*ompC*. Interestingly, the BWΔ*ompF* and the parent strain BW were consistently higher producers of biofilm compared with any strain tested. FHM6 produced more biofilm than most ST131 isolates at both 37°C and 30°C, whereas CUCM247 showed very low biofilm formation and demonstrated no infectivity in either mouse bladder or kidney tissues (Fig. 1).

### Cell adhesion and invasion assays

**Fig 4:**
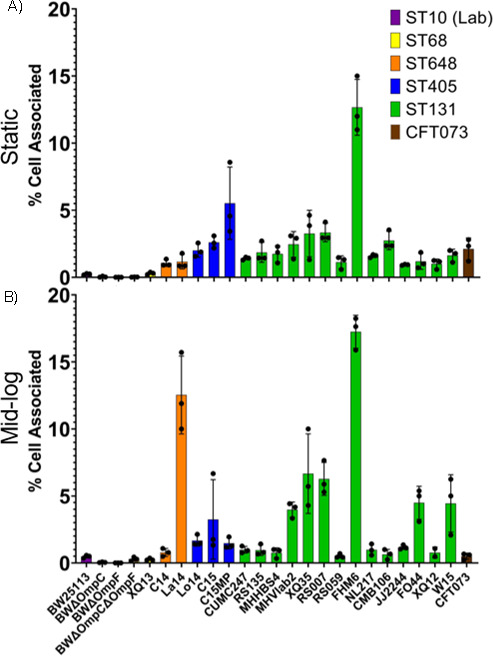
Adherence to T24 human epithelial cells. Epithelial cell adhesion was measured by determining CFUs adherent to epithelial cells as a percentage of inoculum in static (A) and midlog (B) conditions. Isolate sequence type is noted by bar color; bars represent the mean adherence of three biological replicates indicated by data points, and error bars represent standard deviations.

The ability of an *E. coli* cell to attach to bladder cells is one hallmark of pathogenicity. Therefore, we evaluated the ability of the *E. coli* isolates to attach or invade T24 human bladder epithelial cells when *E. coli* were grown under static conditions or grown to mid-log immediately prior to co-culture. Overall, in midlog conditions, the ST131 strains showed greater adherence than non-ST131 strains (3.56% vs. 1.935% respectively, *P* = 0.0897); however, these findings did not reach the threshold for statistical significance. This finding was similar under static conditions with ST131 strains showing statistically significantly more adherence (2.64% vs 1.37%, respectively, *P* = 0.0263). Laboratory BW strains were unable to adhere to epithelial cells under either static or exponential growth conditions (Fig. 4A and B). Isolates C15MP (ST405, 5.5%) and FHM6 (ST131, 12.7%) showed the highest percentage of adherence in static conditions. When grown to mid-log phase prior to coculture, several, but not all, clinical isolates exhibited 1.5-fold to 2-fold higher levels of adhesion to epithelial cells. La14 (ST648) and FHM6 (ST131) showed the most increase in adhesion under mid-log conditions (1.2% to 12.6% and 12.7% to 17.3%, respectively), whereas C15MP (ST405), RS059 (ST131), and CFT073 (ST73) showed 2-fold to 4-fold lower adhesion abilities when grown to mid-log phase before co-culture. FHM6 showed the highest levels of adhesion, followed by C15MP in static conditions and La14 in mid-log cultures, despite the inability of either C15MP or La14 to cause tissue infections in the mouse model, whereas FHM6 effectively colonized both types of tissue.

**Fig 5:**
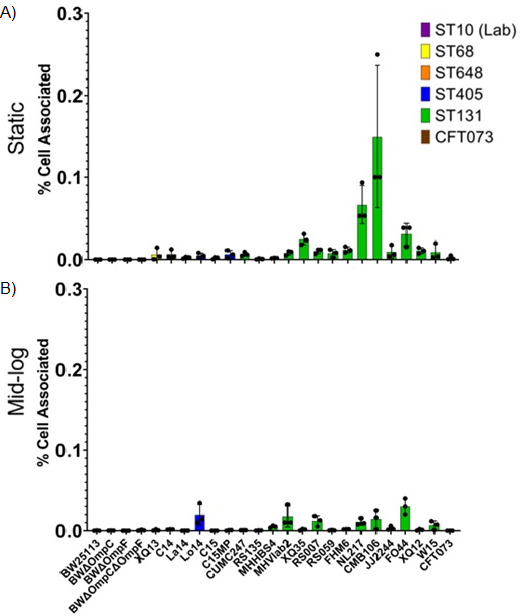
Invasion of T24 human epithelial cells. Percent of bacteria that invaded T4 bladder epithelial cells was measured by determining CFUs of bacteria present in epithelial cell cultures after incubation with Polymyxin B, which killed adherent bacteria. Bacteria that invaded epithelial cells are recorded as a percentage of inoculum in static (A) and mid-log (B) conditions. Isolate sequence type is noted by bar color; bars represent the mean invasion percentage of three biological replicates indicated by data points, and error bars represent standard deviations.

To determine cellular invasion, the same protocol for adhesion was followed with an additional incubation step at the end adding Polymyxin B to the culture media to kill off any bacteria adhered to the outside of the cell. Figure 5 shows a smaller percentage of bacteria were able to invade cells compared with the adherence seen in Fig. 4. Overall, ST131 strains under static conditions showed more invasion than non-ST131 strains (0.02485% vs 0.002763%, respectively, *P* = 0.00224). This finding was similar for midlog conditions with ST131 strains having significantly greater invasion than non-ST131 strains (0.0075% vs. 0.0021% respectively, *P* = 0.00569). Laboratory BW Strains were unable to invade (Fig. 5A and B) epithelial cells under either static or exponential growth conditions. Cells grown statically showed more invasion overall than cells grown to mid-log phase with ST131 isolates showing an average of 9-fold more invasion under both conditions compared with non-ST131 isolates. ST131 isolates CMB106 (0.15%) and NL217 (0.07%) showed the highest percentage of invasion in static conditions, followed by FO44 (0.03%) and XQ35 (0.02%). When grown to mid-log, most *E. coli* clinical isolates exhibited reduced or no invasion into epithelial cells, especially CMB106 (0.01%) and NL217 (0.01%). Lo14 (ST405) showed increased invasion when grown to mid-log phase (0.005% to 0.02%), and FO44 showed similar invasion at 0.03% for both growth conditions. CMB106 and NL217 demonstrated the highest percent of epithelial cell invasion (0.15% and 0.06%, respectively) but were only average compared with all tested ST131s for bladder (50% and 50%, respectively) or kidney (67% and 53%, respectively) colonization in the mouse model.

### Hemagglutination assays

**Fig 6:**
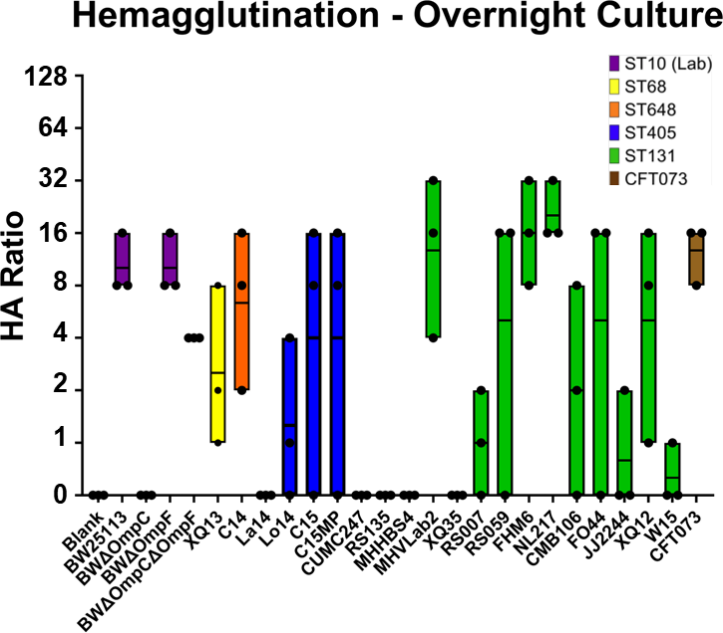
Mannose-sensitive hemagglutination. Hemagglutination assays were performed using guinea pig erythrocytes, and 2-fold serial dilutions as indicated by the HA ratio. Isolate sequence type is noted by bar color; line represents the mean hemagglutination ratio of three biological replicates.

Type 1 fimbriae have been implicated as a pathogenic mechanism for UPEC isolates due to the FimH-binding affinity for uroplakin mannosylated-glycoproteins expressed on urinary tract epithelial cells (31). To determine the ability of the isolates to hemagglutinate red blood cells indicating the use of type 1 fimbriae, mannose-sensitive hemagglutination assays were performed with bacteria grown in both static and mid-log conditions. Under static growth conditions (Fig. 6), ST10 lab strains with the exception of BWΔOmpC and isolates of ST648, ST68, ST405, and CFT073 were all capable of hemagglutination except La14 (Fig. 6; Fig. S6). Four ST131 isolates showed no hemagglutination (CUMC247, RS135, MHHBS4, and XQ35. Interestingly, W15, the most infective isolate in the mouse model, showed very little hemagglutination. However, all isolates showed variability in their ability to hemagglutinate. When isolates were grown to mid-log phase, no isolates were capable of hemagglutination (data not shown). The inclusion of mannose inhibited all isolates from hemagglutination activity (Fig. S6).

### Curli production

**Fig 7:**
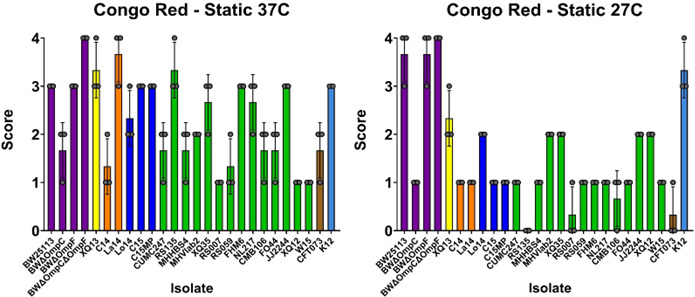
Curli production. Isolates were assessed for curli production in static conditions for either 24 h at 37°C or 48 h at 27°C . Score 0 = an all-white colony, Score 1 = light color, Score 2 = some dark color or ring structures with a matte surface just where the color is, Score 3 = a dark, even color accompanied by a matte/dry surface, and Score 4 = dark color and a rough, wrinkled colony. Isolate sequence type is noted by bar color; bars represent the mean score of three biological replicates indicated by data points, and error bars represent standard deviations. Color coding as indicated in legend for Fig. 1.

The fimbrial adhesin, Curli, encoded by the *csgA* gene is considered a virulence mechanism for uropathogenic *E. coli* due to its role in biofilm formation and cellular adhesion (32). Congo Red assays were used to determine the level of curli production for each isolate evaluated (32). Colonies that remained white produce no curli (Score 0–1), whereas colonies that produce a light color with a matte surface have some curli production (Score 2–3), and isolates with dark-colored colonies and matte or wrinkled surfaces are considered high curli producers (Score 4) (32). Both static and shaking cultures at two different temperatures (37°C and 27°C) were evaluated for curli production (Fig. 7 and Fig. S7). Overall, at 37°C, under shaking growth conditions, the ST131 strains showed less curli production than non-ST131 strains (1.976 vs. 2.933, respectively, *P* = 0.0163) (Fig. S7). This finding was similar under static conditions at 37°C with ST131 strains showing statistically significantly less curli production than non-ST131 strains (1.976 vs 2.833, respectively, *P* = 0.0213). At 27°C, the overall results were consistent; under shaking growth conditions, the ST131 showed less curli production than non-ST131 strains (1.167 vs. 2.067, respectively, *P* = 0.0621). This finding was similar under static conditions at 37°C, with ST131 strains showing less curli production than non-ST131 strains (1.143 vs 2.067, respectively, *P* = 0.056). The lab strain, K12, was a high producer of curli. As the BW lab strains are K12 derivatives, they showed similar levels of curli production to K12, which was attenuated in the OmpC knockout strain. Curli production was variable for clinical isolates of *E. coli,* which as a group showed less curli production for both static and shaking cultures than lab strains at 27°C. For all clinical isolates, there was more curli production at 37°C than 27°C regardless of static or shaking growth condition. It was interesting to note that isolate, RS135, only produced curli when incubated at 37°C in either shaking or static growth conditions. XQ13 produced the most curli of all clinical isolates, despite only infecting one kidney throughout the course of the mouse study. XQ12 and W15 demonstrated higher levels of both bladder and kidney infectivity in the murine model but showed lower levels of curli production.

### Correlations between *in vitro* mechanisms of pathogenesis and *in vivo* infectivity

**Fig 8:**
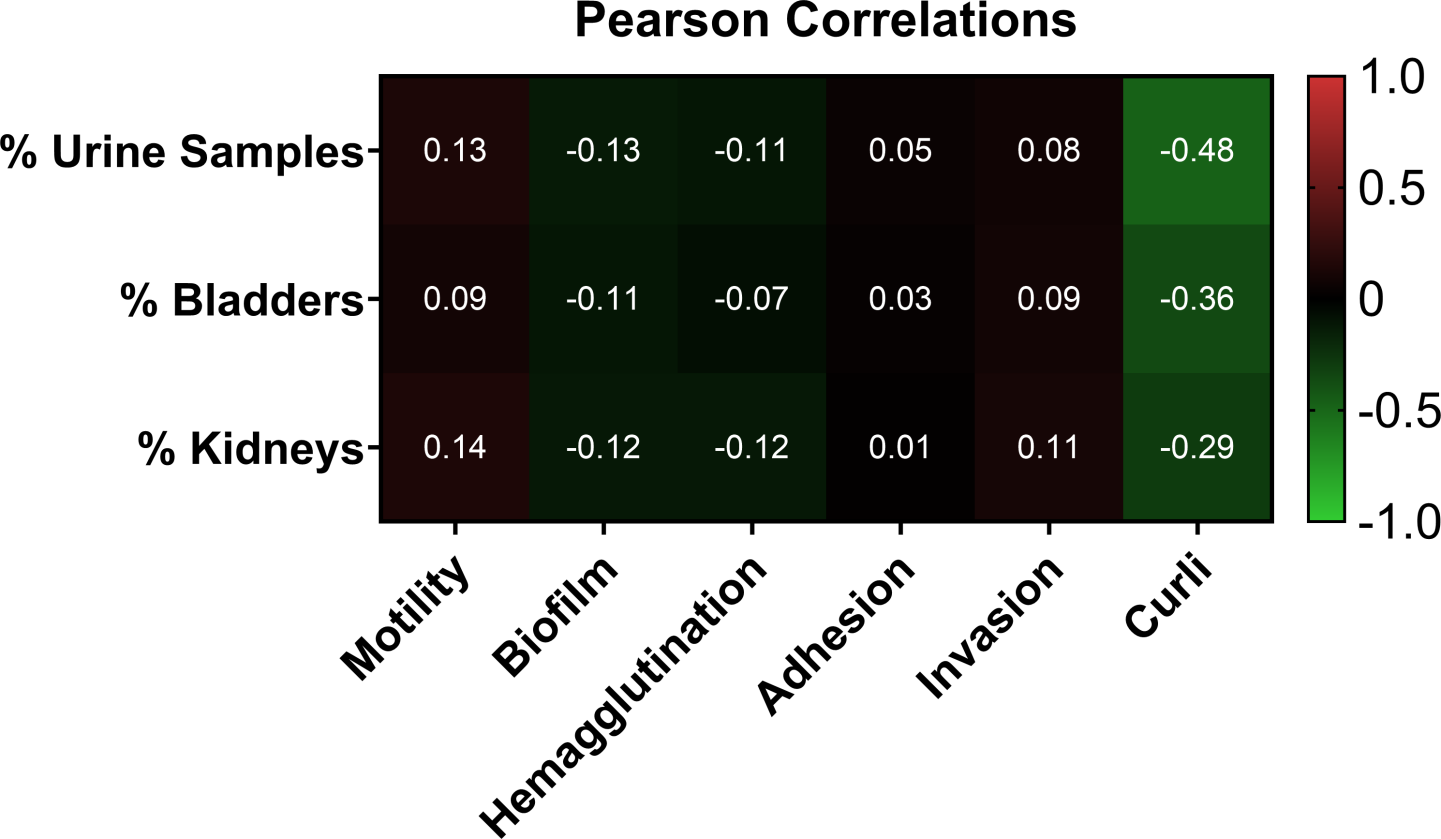
Heatmap Pearson correlations for phenotypic assays and *in vivo* infection results. Heatmap represents Pearson correlations for all independent linear regression (Fig. S7).

Phenotypic *in vitro* assays are thought to recapitulate the infectivity of isolates *in vivo*. To assess the correlation between the *in vitro* and *in vivo* data, we conducted pairwise linear regressions for each assay compared with urine cultures, bladder, and kidney infections (Fig. 8; Fig. S8). Figure S8 shows the correlations for the motility assay compared with the CFUs found in urine, bladder, and kidney during infection. Although the trendline shows a weak positive correlation, the *R*^2^ value for the Pearson correlation is less than 0.14 for any pair showing little to no statistical association. The correlations for hemagglutination compared with urine, bladder, and kidney show a weak negative correlation. However, the *R*^2^ value for the Pearson correlation was less than 0.15 for any pair showing little to no statistical association. The correlations for epithelial adhesion compared with urine, bladder, and kidney show a weak positive correlation, similar to epithelial invasion M-O. However, the *R*^2^ value for the Pearson correlation is less than 0.1 for any pair showing little to no statistical association. Although the sample variance was higher for the curli assay, the Pearson correlation values were the strongest of any phenotypic assay with a negative correlation between curli production and kidney infection (0.2477), (bladder (0.2425), and urine (0.4162)). Figure 8 shows a heatmap corresponding to the strength of the Pearson correlations for the *in vitro* assays compared with the *in vivo* mouse infection results. These results show no statistically significant relationships between any comparisons.

## DISCUSSION

Mouse models of urinary tract infection are an established way to evaluate a host immune response and bacterial pathogenicity to aid in studies to improve human health (16). However, animal studies come with several drawbacks including time, cost, and animal lives. To gain an understanding of bacterial fitness and pathogenicity, without the drawback of *in vivo* experiments, many researchers turn to *in vitro* assessments. For uropathogenic *E. coli*, *in vitro* experiments commonly include motility assays, biofilm production, curli production, adhesion/invasion assays, hemagglutination assays, and detection of bacterial enzymes like β-lactamases (22, 24). How well these experiments predict the virulence of bacteria compared with a mouse model for UTIs has not been determined for the pandemic clone, *E. coli* ST131. Further compounding this problem is the fact that many studies are conducted using common strains of pathogenic or avirluent bacteria such as CFT073, UTI89, or MG1655 and not enough consideration has been given to clinical isolates of bacteria that each possess their own unique characteristics (33–35). In addition, *in vivo* assays do not typically take into account the physiology of cells during an infection. In the establishment of a bladder infection, clusters of cell division-arrested filaments occur in addition to highly motile bacteria (25–28). These observations suggest the presence of subpopulations of *E. coli* during an infection, consisting of replicating and metabolically active cells in addition to static cells. Therefore, static cultures may represent a subpopulation of the infection but not the entire population involved in urinary tract infections. Due to the presence of subpopulations of UPEC during infection, we not only evaluated the *in vitro* assays using static cultures but also evaluated cells from midlog shaking cultures to represent the physiology of the cells more closely during an infection.

Fitness costs have been associated with β-lactamase gene expression (36, 37). Many sequence type 131 *E. coli* carry the extended-spectrum β-lactamase, CTX-M-15, which has been associated with fitness cost (37). RNA expression of *bla*_CTX-M-15_ in ST131 *E.coli* is extremely variable ranging from 1-fold to 165-fold compared with the expression of *bla*_CTX-M-14_ in a ST405 *E. coli* (24). It is interesting to note that strain XQ12 caused pyelonephritis in 90% of animals, whereas strain RS135 was not detected in the urine, bladder, or kidney (Fig. 1). These two strains differed in their level of *bla*_CTX-M-15_ RNA expression by ~8-fold with strain RS135 having the highest level of *bla*_CTX-M-15_ expression but not able to sustain infection in the urinary tract of the mice. This association between infectivity and CTX-M-15 expression was also noted for CUMC247 (no infection and 3.5-fold higher RNA), whereas CMB106 and JJ2244 were capable of infecting both bladder and kidney tissue in 70% and 80% of the mice but differed only by 1.5-fold to 2.5-fold in *bla*_CTX-M-15_ RNA expression, respectively. Thus, the expression level of *bla*_CTX-M-15_ could play a role in the successful infection of the urinary tract by ST131 *E. coli*. A recent study identified differential gene expression of 435 genes in *Klebsiella pneumoniae* when *bla*_CTX-M-15_ was overexpressed (38). These data implicate the ability of CTX-M-15 and other β-lactamases to impact cellular physiology beyond antibiotic resistance.

Looking independently at phenotypic assays, clinical isolates showed less motility than nonpathogenic lab strains, but ST131 strains were more motile than other non-ST131 strains. Given that motility has been identified as a mechanism of pathogenesis, it is not surprising that the ST131 isolates demonstrated greater motility compared to other UPEC and laboratory strains (39). However, a similar study comparing UPEC isolates of several sequence types found that multiple isolates were non-motile after 16 h at 30°C with an equal number hypermobile compared with CFT073; however, our study found that most clinical isolates are more motile than CFT073 (22). Shea et al. also found that most clinical isolates tested, with the exception of strain HM6, produced less biofilm than CFT073 at 37°C, whereas in our study, we found CFT073 to be comparable with some of the ST131 clinical isolates yet lower than the lab strains. Biofilm formation was also dependent on the conditions tested (22). These data reflect the variable nature of clinical isolates in phenotypic testing as noted throughout this manuscript. An unexpected finding from this study was the differential effect of temperature and static vs mid-log growth conditions. Static conditions are considered imperative for the production of type 1 fimbriae and the ability of pathogenic *E. coli* to produce biofilm, hemagglutinate, adhere to, and invade host tissues (31). However, in our experiments, biofilm production increased under shaking conditions for some isolates at 37ᵒC. These findings could implicate that other adhesins are required for biofilm production including adherence-associated surface proteins such as OmpA (40).

Most *in vitro* assays to evaluate attachment to epithelial cells are done using static cultures (41). Static cultures were first used to evaluate fimbriae because static cultures produced increased fimbriae production compared with shaking cultures, making it easier to evaluate protein structure (42). We examined the attachment of *E. coli* ST131 by evaluating fimbriae, curli, and p-pilus using *in vitro* assays with cultures grown in both static and shaking conditions. We reasoned that in a urinary tract infection, it would be unlikely that the *E. coli* would exhibit a static phenotype, given the observation of subpopulations during cystitis (25–28). Overall, ST131 clinical isolates were better able to adhere to, and invade, T24 human epithelial cell cultures than non-ST131 isolates, regardless of static or shaking conditions, but non-pathogenic ST10 lab strains showed limited to no ability for either. These results are corroborated by other phenotypic studies wherein the ST131 isolate had one of the highest association percentages of the clinical isolates tested; however, in that study, the lab strain K12 also showed high cell association, whereas our K12-derived lab strains did not (22). The ability of non-ST131 isolates to adhere and colonize, albeit less successfully than ST131 isolates, suggests pathogenic mechanisms may have evolved in ST131 isolates, enabling them to establish a strong pandemic prevalence (12).

Interestingly, in our study, ST131 isolates were variable in their ability to cause hemagglutination, commonly thought to be directly linked to pathogenicity through the expression of type 1 fimbriae in colonizing host tissues and movement through the ureter into the kidney (31). Hemagglutination varied widely among the isolates. Although none of the laboratory strains infected the mice, both BW and BWΔ*ompF* had strong hemagglutination; however, BWΔ*ompC* showed no hemagglutination. This was interesting, as curli production for this strain was also low compared with the other laboratory strains. Of the non-ST131 isolates, only La14 was unable to hemagglutinate cells, whereas the ST131 isolates were variable. ST131 isolates, CUMC247, RS135, MHHBS4, and XQ35 showed no hemagglutination activity. Of these strains, only XQ35 was capable of infecting both the bladder and the kidney. Of interest, isolate W15 was capable of high infectivity in both the bladder and kidney, and it had minor hemagglutination activity. Variable hemagglutination activity has also been observed for other UPEC isolates (22). The variable hemagglutination activity was unexpected and indicates the importance of other adhesins in the pathogenic phenotype of *E. coli* ST131 such as p-fimbriae and curli (31).

ST131 *E. coli* is often discussed as a group with similar traits, such as antibiotic resistance and some virulence factors. However, WGS of ST131 isolates has confirmed that these strains also have differences with respect to both resistance mechanisms and virulence factors. Johnson and colleagues have identified a common set of virulence factors (*fimH, fyuA, malX, usp, ompT, papA, kpsM,* and *iutA*) that are often found in ST131 (43). However, not all ST131 isolates have this same common set. Many genes encoding these virulence factors are found on the chromosome and constitute a part of the core genome of *E. coli*; however, additional copies of these gene operons can be found in some isolates due to horizontal gene transfer, and some lack the specific genes due to deletion events (44). With the average *E. coli* genome consisting of between 4,000 and 5,000 genes, ST131 *E. coli* share a core genome of 3,712 genes with over 22,000 accessory genes identified across isolates (44, 45). With the extreme variability in ST131 *E. coli* genomes, researchers turn to *in vitro* assays to help evaluate the fitness and virulence of these isolates.

The overall objective of this research was to determine the correlation of commonly used *in vitro* phenotypic assays as predictive indicators for bacterial pathogenicity *in vivo*. At first glance, it appeared that hemagglutination and biofilm production had a negative association with pathogenicity, whereas motility, adhesion, and invasion positively correlated with infectivity. However, the statistical analyses of these findings differed. With Pearson correlations confirming the strongest association at a coefficient of 0.14, it is impossible to say that the *in vitro* results are definitively correlated to or predictive of *in vivo* outcomes. Our results are supported by another recent study where investigators determined weak Pearson correlation values for *in vitro* phenotypic assays to predict *in vivo* bladder colonization in a mouse model (22). Between our data set and that of Shea, we had a slightly higher correlation between motility and urine colonization (0.1349 vs 0.08827) (22). This could be due in part that our study focused primarily on ST131 *E. coli*, whereas Shea et al. included only one ST131, and/or we allowed 28 days prior to sacrifice, whereas Shea et al. (22) ended their study at 48 h post-inoculation. Therefore, our mechanisms could point more toward the bacterial requirements necessary to maintain an infection, whereas the other results indicate the necessary mechanisms to establish an infection (22). Taken together, these two studies show that regardless of the *E. coli* isolates being evaluated, *in vitro* assays may be useful in determining phenotypic differences between isolates of *E. coli,* but their usefulness in predicting pathogenicity is limited.

Although the literature tends to evaluate ST131 *E. coli* as a single pandemic clone, it is clear from this study as well as WGS data that these organisms differ in their genomic makeup and their infectivity in a mouse UTI model. Expression of both CTX-M β-lactamases and OmpC porin production have been shown to vary among strains of ST131, suggesting the physiology of these organisms differ (24, 46). Together, these data identify the uniqueness of individual clinical isolates of ST131. Further analyses on this group of *E. coli* are required with emphasis on physiological differences and how those differences impact pathogenesis.

## MATERIALS AND METHODS

### Cell and bacterial culture conditions

T24 human epithelial bladder cells (ATCC HTB-4) were grown in McCoy’s 5A modified medium (Gibco 16–600-082) with 10% fetal bovine serum (FBS) and 1% penicillin/streptomycin antibiotics at 37°C in the presence of 5% CO_2_. All bacteria strains used in this study are listed in Table 1. *E. coli* isolates were grown in Mueller-Hinton Broth (MHB) for growth curves or Luria Broth (LB) for phenotypic analysis. LB contains 10 g of tryptone and sodium chloride and 5 g of yeast extract per liter. MHB is commonly used for antimicrobial susceptibility testing and conforms to the National Committee for Clinical Laboratory Standards recommended guidelines for cation composition (Ca2+ 20–25 mg/L and Mg2+ 10–12.5 mg/L). Static cultures were incubated at 37°C overnight with no motion; shaking cultures were incubated at 37°C in a ThermoForma Orbital Shaker at 155 rpm either overnight or midlog phase as noted by each assay.

### Murine model of urinary tract infection

All animal studies were overseen by the Institutional Animal Care and Use Committee of Creighton University (Protocol #1174). Female C57BL/6 mice aged 42–48 days were purchased from Charles River Laboratories for this study. A minimum of 6 mice were used for each bacterial strain. Animals were co-housed and provided food and water *ad libitum*. To ensure the urinary CFUs measured were due to the bacteria inoculated, prior to the mouse study isolates were subjected to phenotypic analysis (growth on MacConkey selective differential media and MIC measurements with Ampicillin and Cefotaxime). This allowed the comparison of urine isolates collected throughout the study with the original inoculum to ensure the bacteria detected were due to inoculation and not contamination. On the day of inoculation, bacteria were grown in MHB. Upon reaching midlog (0.5 OD_600_), the culture was diluted to a 0.5 McFarland in sterile saline (1 × 10^8^), and the *E. coli* was suspended in 50 µL of saline. This suspension was used as the inoculum. Prior to catheterization, urine was collected from each mouse and plated on blood agar to detect the presence of any pre-existing urine contamination. To initiate ascending urinary tract infections, mice were anesthetized with isoflurane and inoculated via transurethral catheterization as previously described (47). Briefly, catheters were made by attaching polyethylene tubing (BD Intramedic #22–204008 I.D. 0.011 mm × O.D. 0.28 mm) to a Sub-Q needle (Becton Dickinson #305115 26G 5/8”) on a 1 mL Leur-lock syringe (Fisherbrand #14955464). Urine was collected on days 1, 3, 7, 10, 14, 21, and 28 post-inoculation. Collected urine was serially diluted, and 10 µL was plated on LB agar in duplicate to count CFUs. On day 28, mice were euthanized and aseptically dissected. Blood was collected via cardiac puncture with an insulin syringe, photographs of the kidneys were taken *in situ*, and then, the bladder and kidneys were removed. Half of each kidney was immediately placed into a 1.5 mL tube and stored at −80°C for future use. The other half of each kidney and the bladder were placed into individual tubes containing 500 µL PBS and homogenized using zirconia/silica beads (2.3 mm diameter, BioSpec #11079125z). Homogenized tissues were serially diluted, and 10 µL was plated on LB agar square-grid plates in duplicate to determine CFUs. The remaining tissue homogenate was stored at −80°C for future experiments.

### Bacteria strain phenotyping

#### Phenotypic photos and growth curves

Bacteria strains were grown on blood agar plates overnight at 37°C. Colonies were taken from the blood agar plates and inoculated into 95 mL MHB sidearm flasks to a 0.1 OD600. Cultures were incubated at 37°C, shaking, with OD measurements taken at 15-min intervals for 165 min. At 0.5 OD600, samples were taken and serially diluted to determine mid-log CFUs.

#### Motility

Assays to evaluate flagella-mediated swimming motility (stab-inoculation into a soft agar plate) were performed as previously described with modifications (22). Briefly, overnight cultures of each strain grown on blood agar plates were diluted to a 2.0 McFarland in sterile saline, and 10 µL of the suspension was inoculated directly into the center of a motility plate (0.3% bactoagar wt/vol in LB). Bacteria were cultured for 10 h at 37°C, photographs were taken, and the diameter of the zone of growth was measured in mm.

#### Biofilm formation

Biofilm was measured for each isolate in static and shaking conditions as previously described (48). Briefly, bacteria strains were cultured in overnight static conditions or shaking to mid-log phase at 37°C in LB. Cultures were diluted to 0.5 McFarland, and 100 µL of suspension was added to a 96-well plate containing 100 µL of LB. Plates were incubated at 37°C either statically or shaking, respectively, for 24 h. The media was removed, and wells were washed with sterile water to remove non-adherent bacteria. Plates were stained with 200 µL Crystal Violet for 15 min. Crystal violet was removed, and plates were washed three times with sterile water and left upside down to dry overnight. After drying, stained biofilm was solubilized with 200 µL 30% acetic acid for 15 min. Absorbance was read at 575 nm and blank-adjusted. Biofilm production was compared with the absorbance of a well incubated with only LB and classified as strong (OD >2 × ODc), moderate (1.5 × ODc < OD≤2 × ODc), weak (ODc <OD ≤ 1.5×ODc), or absent (OD ≤ODc) (30).

#### Adhesion and invasion

Human bladder epithelium T24 cells (ATCC #HTB-4) were grown to confluence in 24-well plates. Cells were trypsinized and counted using trypan blue and Countess 3 (ThermoFisher #A50298). Using cell counts, a multiplicity of infection of 100 was determined. Bacteria were grown statically or shaking to mid-log in LB and diluted to a 0.5 McFarland in sterile saline. The appropriate volume of suspension was pelleted by centrifugation and resuspended in 1 mL of serum-free, antibiotic-free, media. T24 cell culture media was removed, the cells were washed with PBS, and bacterial suspension media was added and incubated for 2 h at 37°C 5% carbon dioxide. During this time, bacterial inoculum was serially diluted and plated to determine inoculum CFUs. After incubation, media was removed, and the cells were washed three times with PBS to remove any non-adherent bacteria. Epithelial cells were lysed with 0.4% Triton-X 100 in PBS for 30 min at 4°C shaking. The suspension was serially diluted and plated on LB agar for CFUs. Adherent bacteria are reported as percent CFUs of inoculum. For the invasion assay, T24 cells were cultured with static or shaking bacteria at a MOI 100 as described above. Prior to epithelial cell lysis, T24 cells and adherent bacteria were cultured with media containing Polymyxin B (100 µg/mL) for one and a half hours to kill extracellular bacteria (49). Cultures were then washed with PBS, lysed, and plated as described above. Intracellular bacteria are reported as percent CFUs of inoculum.

#### Hemagglutination

For overnight cultures, a single bacterial colony on an LB agar plate was used to inoculate 5 mL of LB medium and was statically incubated at 37°C for 18–24 h. Ten microliters of this culture were used to inoculate fresh 10 mL of LB medium that was incubated for 24 h under the same conditions. After the second 24 h culture (or for cultures grown to midlog), 1 µL of static bacterial cells was gently pelleted and resuspended in 100 µL PBS with or without 4% mannose. Twenty-five microliters of bacterial suspension were serially diluted (2-fold) in a row of 96-well V-bottomed plates (#3897; Corning); each well contained 25 µL of PBS with or without 4% mannose. Twenty-five microliters of 1% guinea pig erythrocytes were added to each well. The plate was gently mixed and incubated overnight at 4°C. The OD_600_ of overnight static cultures = 0.87–1.12. Midlog cells were pelleted to the same OD_600_ to maintain consistent cell numbers for plating. Mannose-sensitive hemagglutination was evaluated as previously described (22, 50).

#### Curli production

Congo red assay plates were made as previously described (32). In brief, plates were made with 15 g/L agarose, 15.5 g/L low-salt LB broth, and a final concentration of 20 mg/L Coomassie brilliant blue and 40 mg/L Congo red. Bacteria were grown overnight in LB broth under either shaking or static conditions at 37°C. Three microliters of each isolate were spot-plated in triplicate onto two different Congo Red plates. One plate was incubated at 37°C for 24 h, whereas the other was incubated for 48 h at 27°C. Colony color and morphology were scored as previously described by three independent evaluators and then aggregated. Colonies were scored as follows: Score 0 = an all-white colony, Score 1 = light color, Score 2 = some dark color or ring structures with a matte surface just where the color is, Score 3 = a dark, even color accompanied by a matte/dry surface, and Score 4 = dark color and a rough, wrinkled colony (32).

### Statistical analysis

Data are presented as mean values of three replicates. Statistical analyses were conducted in GraphPad Prism version 9.5.1. For phenotypic assays and bacterial load, one-way analysis of variance (ANOVA) with post-hoc Tukey tests was conducted to compare differences between strains. Differences between ST131 and Non-ST131 group results for *in vitro* assays were determined using two-tailed Welch’s *t*-test. For mouse studies, proportions were compared using two-tailed Fisher’s exact tests. Pearson correlations were conducted between all phenotypic and *in vivo* data sets individually and compared using multiple linear regressions. The threshold for statistical significance for all analyses was set at *P* ≤ 0.05.

## SUPPLEMENTAL MATERIAL

Figure S1 spectrum.01511-24-s0001.tif Growth curves for all strains used in this study.

Figure S2(A) spectrum.01511-24-s0002.tif Urine colonization over time - all strains.

Figure S2(B-E) spectrum.01511-24-s0003.tif Urine colonization over time - select strains.

Figure S3 spectrum.01511-24-s0004.tif Motility assay plate photographs.

Figure S4 spectrum.01511-24-s0005.tif Biofilm assay 30°C Midlog Static.

Figure S5 spectrum.01511-24-s0006.tif Biofilm assay 30°C Midlog shaking.

Figure S6 spectrum.01511-24-s0007.tif Hemagglutination assay plate images.

Figure S7 spectrum.01511-24-s0008.tif Curli assay shaking, 37°C and 27°C.

Figure S8 spectrum.01511-24-s0009.tif Individual assay Pearson correlations.

Legends spectrum.01511-24-s0010.docx Supplemental Figure Legends.

Table S1 spectrum.01511-24-s0011.tif Growth Curve OD Readings from Figure S1.

ASM does not own the copyrights to Supplemental Material that may be linked to, or accessed through, an article. The authors have granted ASM a non-exclusive, world-wide license to publish the Supplemental Material files. Please contact the corresponding author directly for reuse.
