# Supplementary figures and images for: In vitro assays for clinical isolates of sequence type 131 Escherichia coli do not recapitulate in vivo infectivity using a murine model of urinary tract infection
Source: Microbiol Spectr. 2025 Feb 25;13(4):e01511-24. doi: 10.1128/spectrum.01511-24 (PMC11960073; doi:10.1128/spectrum.01511-24)

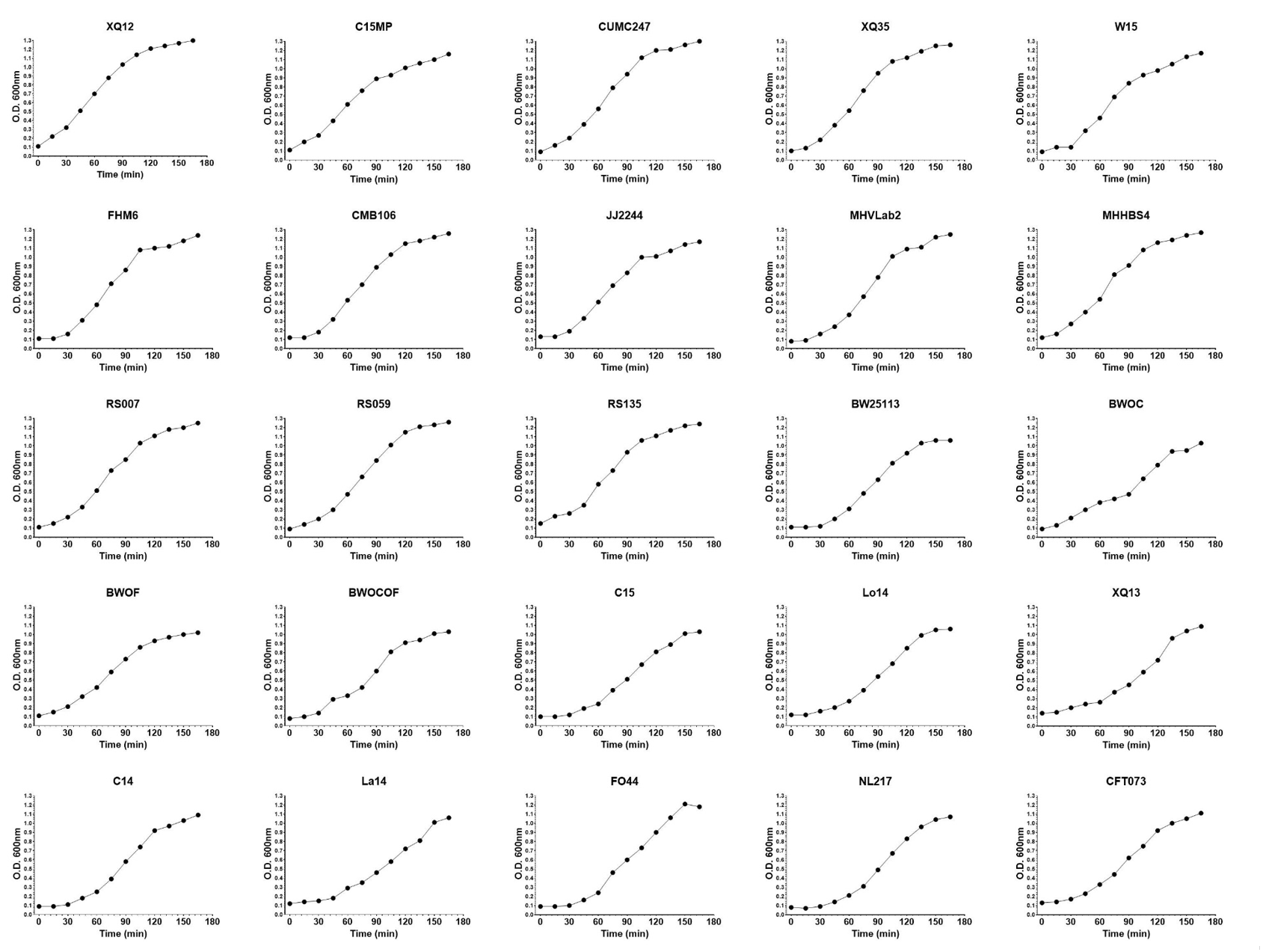

Supplement: Figure S1 — Growth curves for all strains used in this study. [file spectrum.01511-24-s0001.tif]

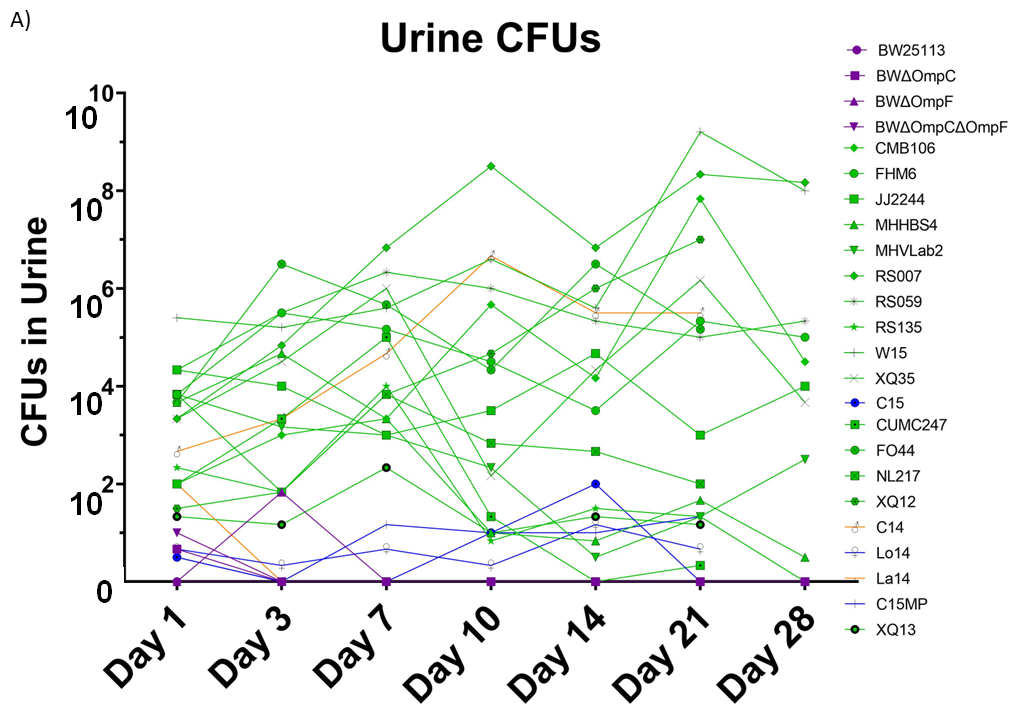

Supplement: Figure S2(A) — Urine colonization over time - all strains. [file spectrum.01511-24-s0002.tif]

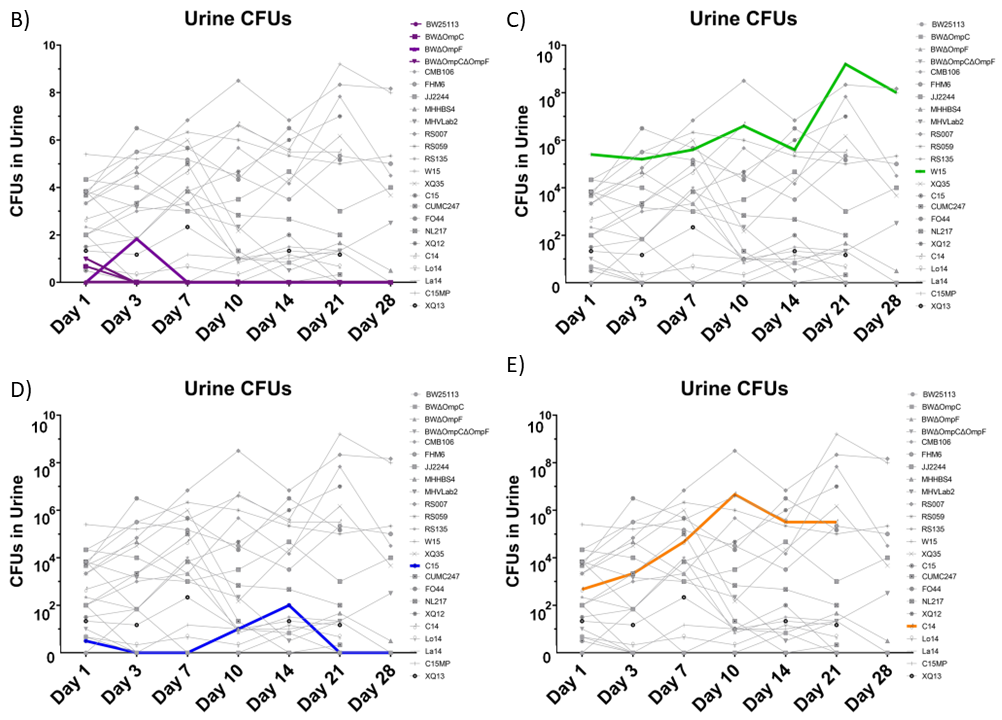

Supplement: Figure S2(B-E) — Urine colonization over time - select strains. [file spectrum.01511-24-s0003.tif]

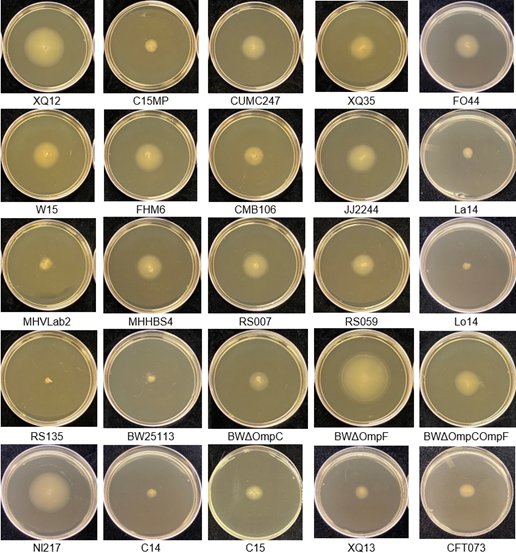

Supplement: Figure S3 — Motility assay plate photographs. [file spectrum.01511-24-s0004.tif]

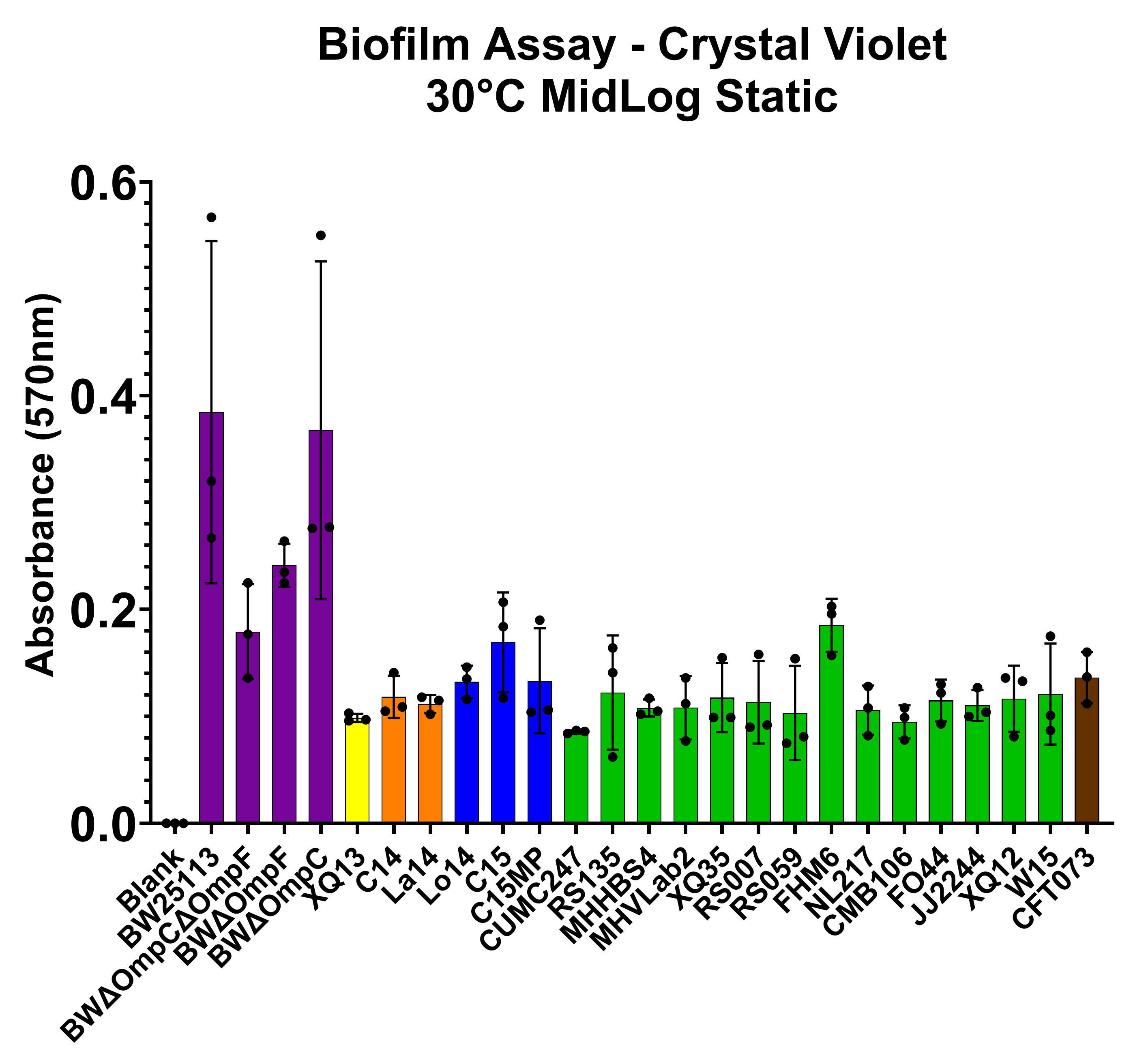

Supplement: Figure S4 — Biofilm assay 30°C Midlog Static. [file spectrum.01511-24-s0005.tif]

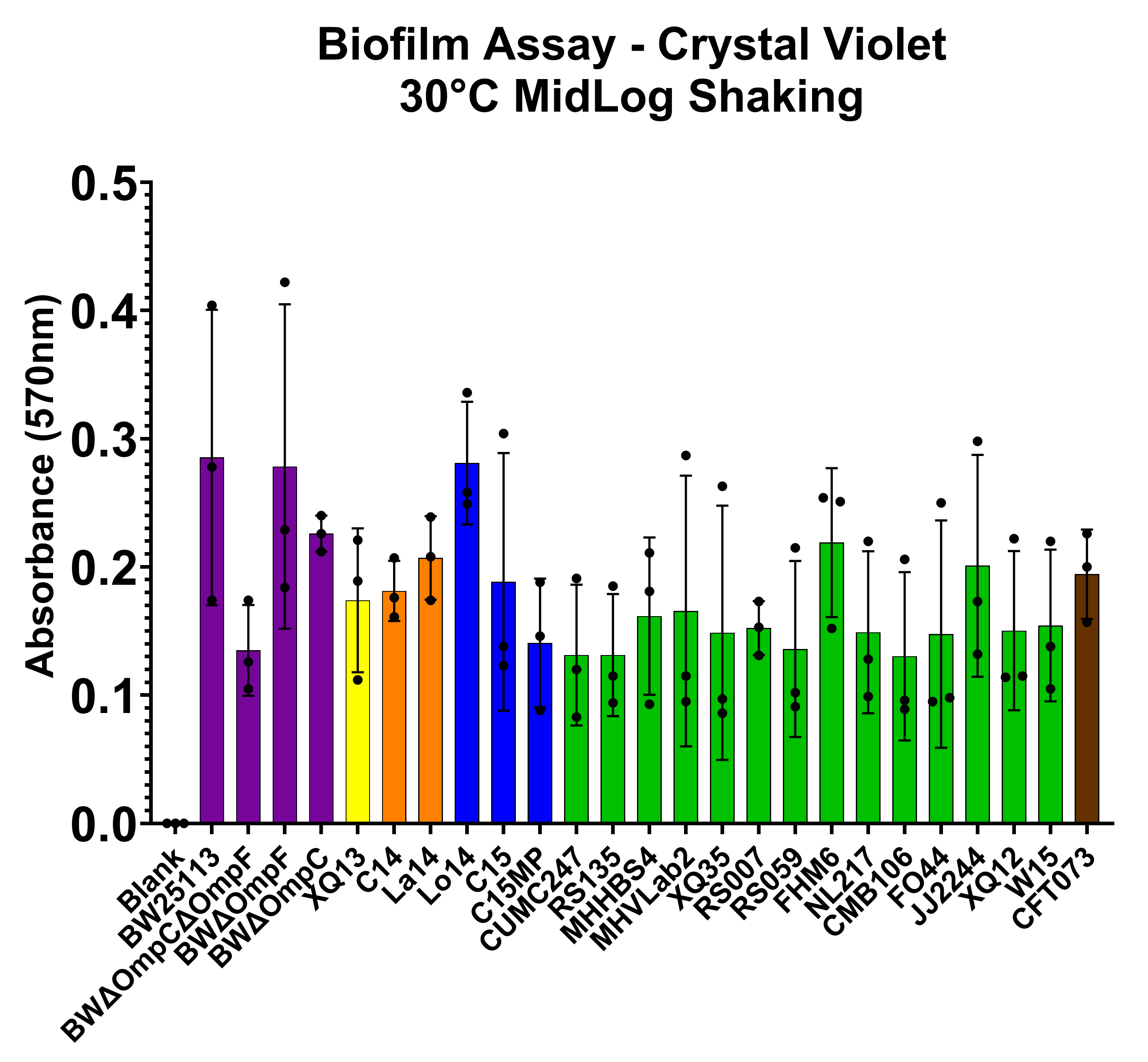

Supplement: Figure S5 — Biofilm assay 30°C Midlog shaking. [file spectrum.01511-24-s0006.tif]

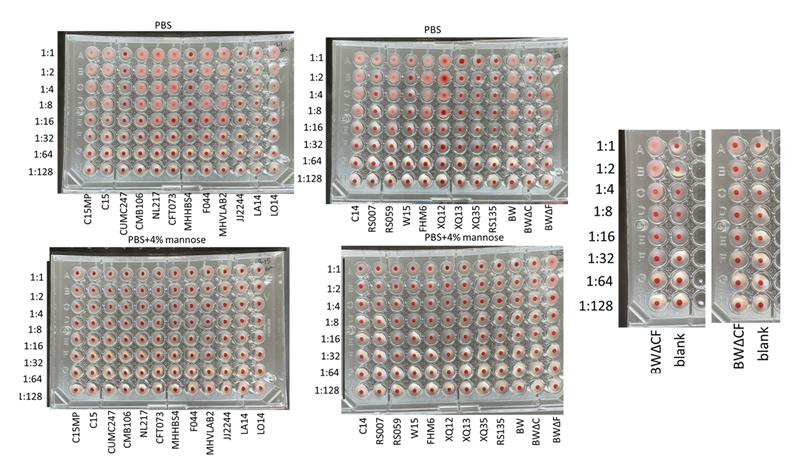

Supplement: Figure S6 — Hemagglutination assay plate images. [file spectrum.01511-24-s0007.tif]

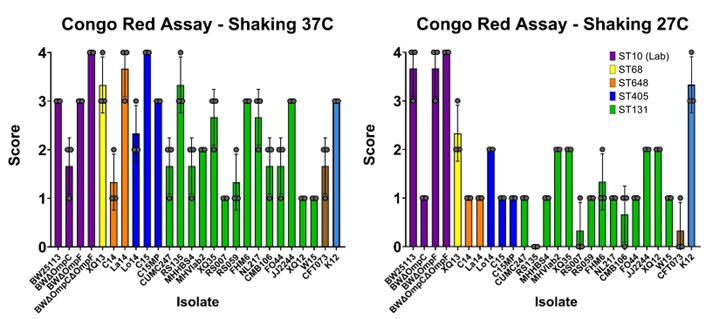

Supplement: Figure S7 — Curli assay shaking, 37°C and 27°C. [file spectrum.01511-24-s0008.tif]

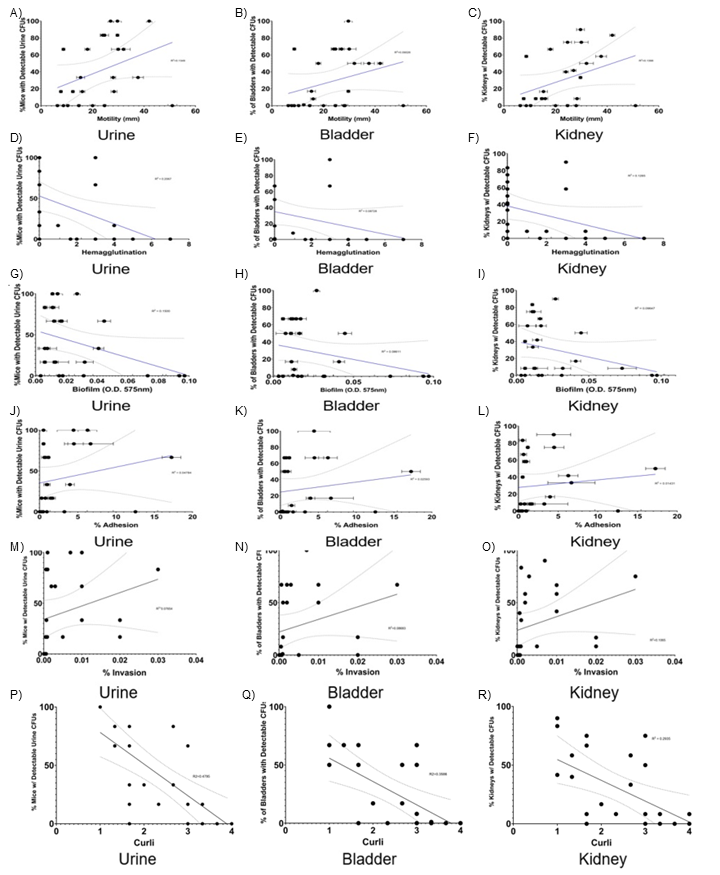

Supplement: Figure S8 — Individual assay Pearson correlations. [file spectrum.01511-24-s0009.tif]

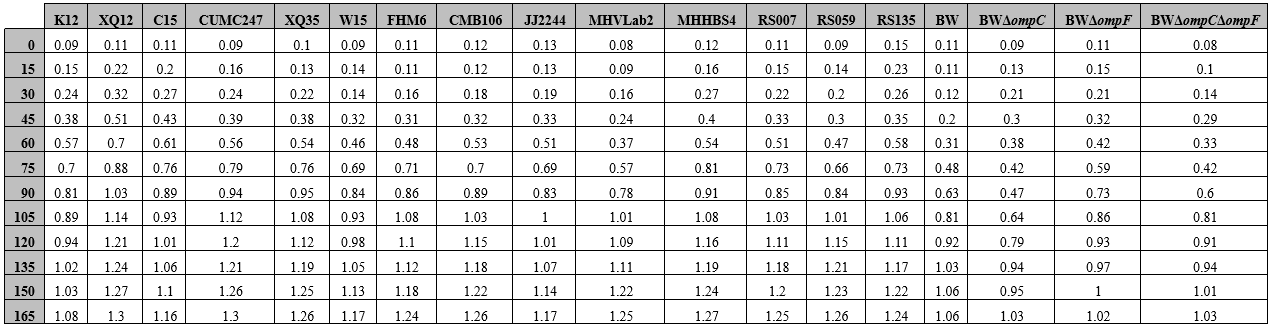

Supplement: Table S1 — Growth Curve OD Readings from Figure S1. [file spectrum.01511-24-s0011.tif]
