# Supplementary material for: In vitro assays for clinical isolates of sequence type 131 Escherichia coli do not recapitulate in vivo infectivity using a murine model of urinary tract infection
Source: Microbiol Spectr. 2025 Feb 25;13(4):e01511-24. doi: 10.1128/spectrum.01511-24 (PMC11960073; doi:10.1128/spectrum.01511-24)
Supplement: Legends — Supplemental Figure Legends. [file spectrum.01511-24-s0010.docx]

**Supplemental Figure Legends**

**Supplemental Figure 1. Growth curves for all *E. coli* isolates used in this study.** (SF1A) Growth curves are similar among isolates and the growth of the organisms do not account for the differences in phenotypic results or mouse model infectivity. O.D.: Optical Density at 600nm. Table of doubling times in minutes as noted by O.D. 600nm reading for each isolate represented in supplemental table 1.

**Supplemental Table 1. Optical Density Readings for Growth curves of all *E. coli* isolates used in this study.** Doubling times in minutes as noted by O.D. 600nm reading for each isolate represented in Figure S1.

**Supplemental Figure 2. Urine Colonization.** Urine was collected to determine colony forming units. All isolates (FigS2A). SF 2B) BWΔ*ompF*. (SF 2C) W15. (SF 2D) C15. (SF 2E) C14. Data points represent the mean CFUs of all urine samples collected for a specific isolate at each timepoint. Graph line and point color represent isolate sequence type as noted in the figure legend.

**Supplemental Figure 3. Motility Assay Plate Photographs.** Motility assays were performed by inoculating soft agar plates (0.3% bactoagar w/v in LB) with a 10µL solution of overnight cultures of each strain which were grown on blood agar plates and diluted to a 2.0 McFarland in sterile saline. Plates were incubated for 10 hours at 37°C and then zone of growth diameter was measured in mm. Measurements for each isolate are listed in Figure 2.

**Supplemental Figure 4**. Biofilm assay using cells grown to midlog and then allowed to form biofilms at 30ᵒC statically. Overnight cultures diluted 1:100 in 5ml fresh LB broth and allowed to grow to OD_600_ = 0.5 at 37°C. Cultures grown to midlog were diluted by adding 500ml to 3ml fresh LB broth to reach a McFarland of 0.15 – 0.21. 1.5ml transferred into 24-well tissue culture plates (TPP #92024) and incubated under static conditions.

**Supplemental Figure 5**. Biofilm assay using cells grown to midlog and then allowed to form under shaking conditions (100 rpms) at 30ᵒC. Overnight cultures diluted 1:100 in 5ml fresh LB broth and allowed to grow to OD_600_ = 0.5 at 37°C. Cultures grown to midlog were diluted by adding 500ml to 3ml fresh LB broth to reach a McFarland of 0.15 – 0.21. 1.5ml transferred into 24-well tissue culture plates (TPP #92024) and incubated under static conditions at 30°C.

**Supplemental Figure 6. Hemagglutination assays**. Assays were completed in the presence and absence of mannose as indicated as described in the methods.

**Supplemental Figure 7. Curli Production.** Isolates were assessed for curli production in shaking conditions for either 24 hours at 37°C or 48 hours at 27°C Score 0 = an all-white colony, Score 1 = light color, Score 2 = some dark color or ring structures with a matte surface just where the color is, Score 3 = a dark, even color accompanied by a matte/dry surface, Score 4 = dark color and a rough, wrinkled colony. Isolate sequence type is noted by bar color, bars represent the mean score of three biological replicates indicated by data points and error bars represent standard deviations.

**Supplemental Figure 8. Linear regressions for phenotypic assays and *in vivo* infection results.** Linear regressions of motility assays compared to (SF 8A) urine CFUs, (SF 8B) bladder CFUs, and (SF 8C) kidney CFUs. Linear regressions of hemagglutination assays compared to (SF 8D) Urine CFUs, (SF 8E) Bladder CFUs, and (SF 8F) Kidney CFUs. Linear regressions of biofilm production compared to (SF 8G) Urine CFUs, (SF 8H) Bladder CFUs, and (SF 8I) Kidney CFUs. Linear regressions of T24 epithelial cell adhesion assays compared to (SF 8J) Urine CFUs, (SF 8K) Bladder CFUs, and (SF 8L) Kidney CFUs. Linear regressions of invasion assays compared to (SF 8M) Urine CFUs, (SF 8N) Bladder CFUs, and (SF 8O) Kidney CFUs. Linear regressions of congo red curli assays compared to (SF 8P) Urine CFUs, (SF 8Q) Bladder CFUs, and (SF 8R) Kidney CFUs. Data points represent the mean colonization and phenotypic results for each isolate, error bars indicate 95% confidence intervals for the linear regression.
